# Comparison of vasoactive-inotropic score, vasoactive-ventilation-renal score, and modified vasoactive-ventilation-renal score for predicting the poor prognosis after coronary artery bypass grafting

**DOI:** 10.1186/s12872-023-03313-9

**Published:** 2023-05-24

**Authors:** Yanping Du, Wensu Li, Qingjuan Chen, Haichuan Shi, Qiong Li, Chunying Zhang, Yunxu Zhuang, Junying Li, Li Tang

**Affiliations:** Department of Cardiac Critical Care Medicine, Affiliated Hospital of Jining Medical University, No.89 Guhuai Road, Rencheng District, 272000 Jining, P.R. China

**Keywords:** VIS, VVR, M-VVR, Coronary artery bypass grafting, Poor prognosis

## Abstract

**Background:**

Exploring reliable prediction scoring systems is valuable for the poor prognosis of patients after coronary artery bypass grafting (CABG). Herein, we explored and compared the predictive performance of vasoactive-inotropic score (VIS), vasoactive-ventilation-renal (VVR) score, and modified VVR (M-VVR) score in the poor prognosis of patients undergoing CABG.

**Methods:**

A retrospective cohort study was performed in Affiliated Hospital of Jining Medical University, and data of 537 patients were collected from January 2019 to May 2021. The independent variables were VIS, VVR, and M-VVR. Study endpoint of interest was the poor prognosis. Association between VIS, VVR, M-VVR and poor prognosis was assessed using logistic regression analysis, and odds ratios (OR) and 95% confidence intervals (CIs) were reported. The performance of VIS, VVR, and M-VVR to predict the poor prognosis was assessed by calculating the area under the curve (AUC), and differences of the AUC of the three scoring systems were compared using DeLong test.

**Results:**

After adjusting gender, BMI, hypertension, diabetes, surgery methods, and left ventricular ejection fraction (LVEF), VIS (OR: 1.09, 95%CI: 1.05–1.13) and M-VVR (OR: 1.09, 95%CI: 1.06–1.12) were associated with the increased odds of poor prognosis. The AUC of M-VVR, VVR, and VIS was 0.720 (95%CI: 0.668–0.771), 0.621 (95%CI: 0.566–0.677), and 0.685 (95%CI: 0.631–0.739), respectively. DeLong test displayed that the performance of M-VVR was better than VVR (*P* = 0.004) and VIS (*P* = 0.003).

**Conclusions:**

Our study found the good prediction performance of M-VVR for the poor prognosis of patients undergoing CABG, indicating that M-VVR may be a useful prediction index in the clinic.

**Supplementary Information:**

The online version contains supplementary material available at 10.1186/s12872-023-03313-9.

## Background

Coronary artery bypass grafting (CABG) is a common operation for revascularization through grafting bypass vessels, and has been the standard for the treatment of coronary artery disease (CAD) [1, 2]. It is estimated that 370,000 CABG are performed in the United States annually [3]. A continuously increasing trend of this operation is observed in China [4]. Despite a decrease in the operative complications, operative mortality, and in-hospital mortality due to improvements in surgery technology and nursing quality, in-hospital prognosis for patients undergoing CABG remains a common concern [5–7].

Some prediction scoring systems, such as Acute Physiology and Chronic Health Evaluation (APACHE), European System for Cardiac Operative Risk Evaluation (EuroSCORE), and vasoactive-inotropic score (VIS), have been developed to predict the poor prognosis of patients undergoing CABG [8, 9]. APACHE performs well in the prediction of renal complications, while the performance is not good in the prediction of cardiovascular and respiratory complications [8]. Evidence has showed that vasoactive-inotropic score (VIS) is a more important scoring system than EuroSCORE in predicting the prognosis of patients undergoing CABG [9]. VIS is a numerical scale demonstrating the amount of vasoactive and inotropic support, and has been reported as an effective predictor for mortality and morbidity of infants and adults in the cardiac surgery [10, 11]. Baysal et al. found that VIS can independently predict early postoperative morbidity and mortality in patients undergoing CABG [9]. Due to the heterogeneity of patients’ anatomy and pathophysiology, vasoactive-ventilation-renal (VVR) score is reported [12]. VVR score is a novel disease severity index based on VIS, and calculated as ventilation index (VI) + VIS + Δ creatinine (ΔCr) × 10 [13], which incorporates the markers of cardiovascular, pulmonary and renal function and has been reported to outperform VIS in predicting hospital-stay following congenital heart surgery [12–14]. In CABG, the predictive value of VVR has not been reported.

Dr Colombo points out that assessing renal dysfunction in VVR score by calculating ΔCr is inaccurate, and creatinine clearance (Ccr) is a more reliable marker [15]. Evidence has shown Ccr is an effective marker to predict renal dysfunction [16, 17]. Therefore, our study modifies the calculation of VVR, and uses Ccr to replace ΔCr in the former formula, which called as modified VVR (M-VVR).

In this study, we aimed to explore the predictive value of M-VVR, VVR, and VIS in the in-hospital prognosis after CABG, and to compare the prediction performance of these three scoring systems.

## Methods

### Study design and study population

This retrospective cohort study was performed in the Affiliated Hospital of Jining Medical University from January 2019 to May 2021, and has been approved by the Ethics Committee of Affiliated Hospital of Jining Medical University (2021C165). All participants have provided the informed consent.

Participants who aged 18–80 years (male or female), met the indications for CABG [18] and underwent CABG; surviving over 48 h in the intensive care unit (ICU), and with complete medical records were included in our study. Those who met one of the following criteria were excluded: (1) undergoing other concomitant cardiac surgeries; (2) with acute myocardial infarction within 30 days before the surgery; (3) previously taking hormonotherapy and immunosuppressant therapy; (4) with malignant tumors or immune diseases; (5) with infectious diseases before the surgery; (6) with severe hepatic and renal insufficiency before the surgery; (7) with valvular heart disease and other heart diseases; (8) not the first time to undergo coronary artery intervention.

### Independent variables

VIS, VVR and M-VVR were independent variables and data at postoperative 24 h were collected. The calculation formulas of VIS, VVR and M-VVR were shown in Supplementary file 1.

### Study endpoint

The study endpoint was the poor prognosis, including at least one of the following: death, cardiopulmonary resuscitation, mechanical circulatory support, low cardiac output syndrome (LCOS; cardiac index < 2.5 L/min/m^2^), stroke, acute kidney injury (with need for renal replacement therapy), and nervous system damage.

### Potential confounders

The following variables were potential confounders in this study: (1) physical characteristics [gender, age, body mass index (BMI)]; (2) living habits (smoking and drinking); (3) history of diseases (hypertension, diabetes, hyperlipidemia, cerebrovascular disease, chronic nephrosis, chronic obstructive pulmonary disease); (4) comorbidity (no/yes); (5) surgery methods (off-pump and on-pump); and (6) left ventricular ejection fraction (LVEF). LVEF was categorized into poor (< 30%), moderate (30-50%), normal (> 50%) groups according to previous study [19].

### Sample size estimation

VVR ≥ 35 were taken as the predictor for mortality to calculate the sample size (OR: 4.95) [12]. α was equal to 0.05, and β was equal to 0.1. Exposure rate of control group was 0.02. The estimated sample size was 214 in each group, calculated by the power analysis software PASS V.15 (NCSS, Kaysville, Utah, USA). Considering a dropout rate of 20%, at least 535 participants were needed.

### Statistical analysis

Continuous variables in normal distribution were expressed as mean ± standard deviation (Mean ± SD), and differences between two groups were compared using t test. Continuous variables in skew distribution were expressed as median and interquartile range [M (Q1, Q3)], and differences between two groups were compared using Mann-whitney U test. Counting data were expressed as number (n) and percentage (%), and differences between two groups were compared using chi-square test.

Univariate and multivariable logistic regression analysis was used to explore the association between VIS, VVR, M-VVR and poor prognosis, and odds ratios (OR) and 95% confidence intervals (CIs) were reported. In the multivariable logistic regression model, gender, BMI, hypertension, diabetes, surgery methods, and LVEF were adjusted. Receiver operating characteristics (ROC) curves of VIS, VVR, and M-VVR predicting the poor prognosis were generated using R version 4.0.3 (The R Foundation for Statistical Computing, Vienna, Austria). Prediction performance of VIS, VVR, M-VVR was assessed by calculating the area under the curve (AUC), with 95%CI. DeLong test was used to compare differences of the AUC of VIS, VVR, and M-VVR. The calibration of the scoring system was assessed using Hosmer-lemeshow goodness of fit test.

To further verify the predictive performance of M-VVR, we compared the prediction ability of M-VVR with that of Sino System for Coronary Operative Risk Evaluation (SinoSCORE), which was divided into three groups according to the risk scores (≤ 1, 2–5, ≥ 6) [20]. *P* value less than 0.05 was considered statistically significant. Statistical analyses were performed using SAS 9.4 (SAS Institute Inc., Cary, NC, USA) and R (version 4.0.3).

## Results

### Patient characteristics

A total of 537 patients undergoing CABG were finally included in our study according to the inclusion and exclusion criteria, and the poor prognosis occurred in 133 of the patients. The number and percentage of each complication were shown in Supplementary table S1. There were 66.48% of male (n = 357) and 33.52% of female (n = 180). The mean age was 65.39 ± 7.66 years and mean BMI was 24.97 ± 3.22 kg/m^2^. Gender, BMI, hypertension, diabetes, surgery methods, LVEF, VIS, VVR, and M-VVR were statistically different between the two groups (all *P* < 0.05) (Table 1).

**Table 1:** Characteristics of the study patients

Variables	Total (n = 537)	Poor outcome		Statistics	*P*
		No (n = 404)	Yes (n = 133)		
Gender, n (%)				χ^2^ = 4.117	0.042
Male	357 (66.48)	259 (64.11)	98 (73.68)		
Female	180 (33.52)	145 (35.89)	35 (26.32)		
Age (year), mean ± SD,	65.39 ± 7.66	65.78 ± 7.25	64.20 ± 8.71	t = 1.89	0.060
BMI (kg/m^2^), mean ± SD	24.97 ± 3.22	24.75 ± 3.19	25.64 ± 3.21	t = -2.78	0.006
Smoking, n (%)				χ^2^ = 0.841	0.359
No	317 (59.03)	243 (60.15)	74 (55.64)		
Yes	220 (40.97)	161 (39.85)	59 (44.36)		
Drinking, n (%)				χ^2^ = 1.279	0.258
No	358 (66.67)	264 (65.35)	94 (70.68)		
Yes	179 (33.33)	140 (34.65)	39 (29.32)		
Hypertension, n (%)				χ^2^ = 8.889	0.003
No	204 (37.99)	139 (34.41)	65 (48.87)		
Yes	333 (62.01)	265 (65.59)	68 (51.13)		
Diabetes, n (%)				χ^2^ = 5.582	0.018
No	356 (66.29)	279 (69.06)	77 (57.89)		
Yes	181 (33.71)	125 (30.94)	56 (42.11)		
Hyperlipidemia, n (%)				χ^2^ = 0.170	0.680
No	516 (96.09)	389 (96.29)	127 (95.49)		
Yes	21 (3.91)	15 (3.71)	6 (4.51)		
Cerebrovascular disease, n (%)				χ^2^ = 0.309	0.578
No	425 (79.14)	322 (79.70)	103 (77.44)		
Yes	112 (20.86)	82 (20.30)	30 (22.56)		
Chronic nephrosis, n (%)				χ^2^ = 3.005	0.083
No	527 (98.14)	399 (98.76)	128 (96.24)		
Yes	10 (1.86)	5 (1.24)	5 (3.76)		
Chronic obstructive pulmonary disease, n (%)				χ^2^ = 0.745	0.388
No	526 (97.95)	397 (98.27)	129 (96.99)		
Yes	11 (2.05)	7 (1.73)	4 (3.01)		
Comorbidity, n (%)				χ^2^ = 2.022	0.155
No	275 (51.21)	214 (52.97)	61 (45.86)		
Yes	262 (48.79)	190 (47.03)	72 (54.14)		
Surgery methods, n (%)				χ^2^ = 5.169	0.023
Off-Pump	296 (55.12)	234 (57.92)	62 (46.62)		
On-Pump	241 (44.88)	170 (42.08)	71 (53.38)		
LVEF (%), n (%)				χ^2^ = 31.711	< 0.001
Poor (< 30%)	67 (12.48)	47 (11.63)	20 (15.04)		
Moderate (30-50%)	93 (17.32)	50 (12.38)	43 (32.33)		
Normal (> 50%)	377 (70.20)	307 (75.99)	70 (52.63)		
VIS, M (Q_1_, Q_3_)	0.00 (0.00,4.00)	0.00 (0.00,0.00)	4.10 (0.00,6.19)	Z = 7.562	< 0.001
VVR, M (Q_1_, Q_3_)	8.00 (4.00,14.00)	7.00 (2.00,12.00)	12.00 (6.00,20.00)	Z = 6.410	< 0.001
M-VVR, M (Q_1_, Q_3_)	99.96 (83.97,118.34)	96.91 (82.41,113.14)	108.53 (89.74,133.13)	Z = 4.196	< 0.001
SinoSCORE, n (%)	55.31 ± 8.11	56.52 ± 6.94	51.47 ± 10.14	t = 4.94	< 0.001
≤ 1				χ^2^ = 5.137	0.077
2–5	210 (39.11)	162 (40.10)	48 (36.09)		
≥ 6	119 (22.16)	96 (23.76)	23 (17.29)		

BMI, body mass index; Mean ± SD, mean ± standard deviation; VIS, vasoactive-inotropic score; VVR, vasoactive-ventilation-renal score; M-VVR, modified vasoactive-ventilation-renal score; LVEF, left ventricular ejection fraction; SinoSCORE, Sino System for Coronary Operative Risk Evaluation

### Association of VIS, VVR, and M-VVR with the poor prognosis

In the unadjusted model, VIS (OR: 1.10, 95%CI: 1.07–1.13), VVR (OR: 1.01, 95%CI: 1.00-1.02), and M-VVR (OR: 1.10, 95%CI: 1.07–1.12) were associated with the increased odds of poor prognosis in patients undergoing CABG. After adjusting gender, BMI, hypertension, diabetes, surgery methods, and LVEF, VIS (OR: 1.09, 95%CI: 1.05–1.13) and M-VVR (OR: 1.09, 95%CI: 1.06–1.12) were found to be associated with the poor prognosis. There was no statistical significance between VVR and the poor prognosis, with OR of 1.00 (95%CI: 0.99–1.01) and *P* value of 0.270. The results were shown in Table 2.

**Table 2:** Univariate and multivariate analyses for the association between VIS, VVR, M-VVR and poor prognosis

Variables	Model 1		Model 2	
	OR (95% CI)	*P*	OR (95% CI)	*P*
VIS	1.10 (1.07–1.13)	< 0.001	1.09 (1.05–1.13)	< 0.001
VVR	1.01 (1.00-1.02)	0.009	1.00 (0.99–1.01)	0.270
M-VVR	1.10 (1.07–1.12)	< 0.001	1.09 (1.06–1.12)	< 0.001

VIS, vasoactive-inotropic score; VVR, vasoactive-ventilation-renal score; M-VVR, modified vasoactive-ventilation-renal score; OR, odds ratio; CI, confidence interval

Model 1, unadjusted model;

Model 2, adjusted for gender, BMI, hypertension, diabetes, surgery methods, and LVEF.

### Comparing the performance of VIS, VVR, and M-VVR

**Fig. 1:**
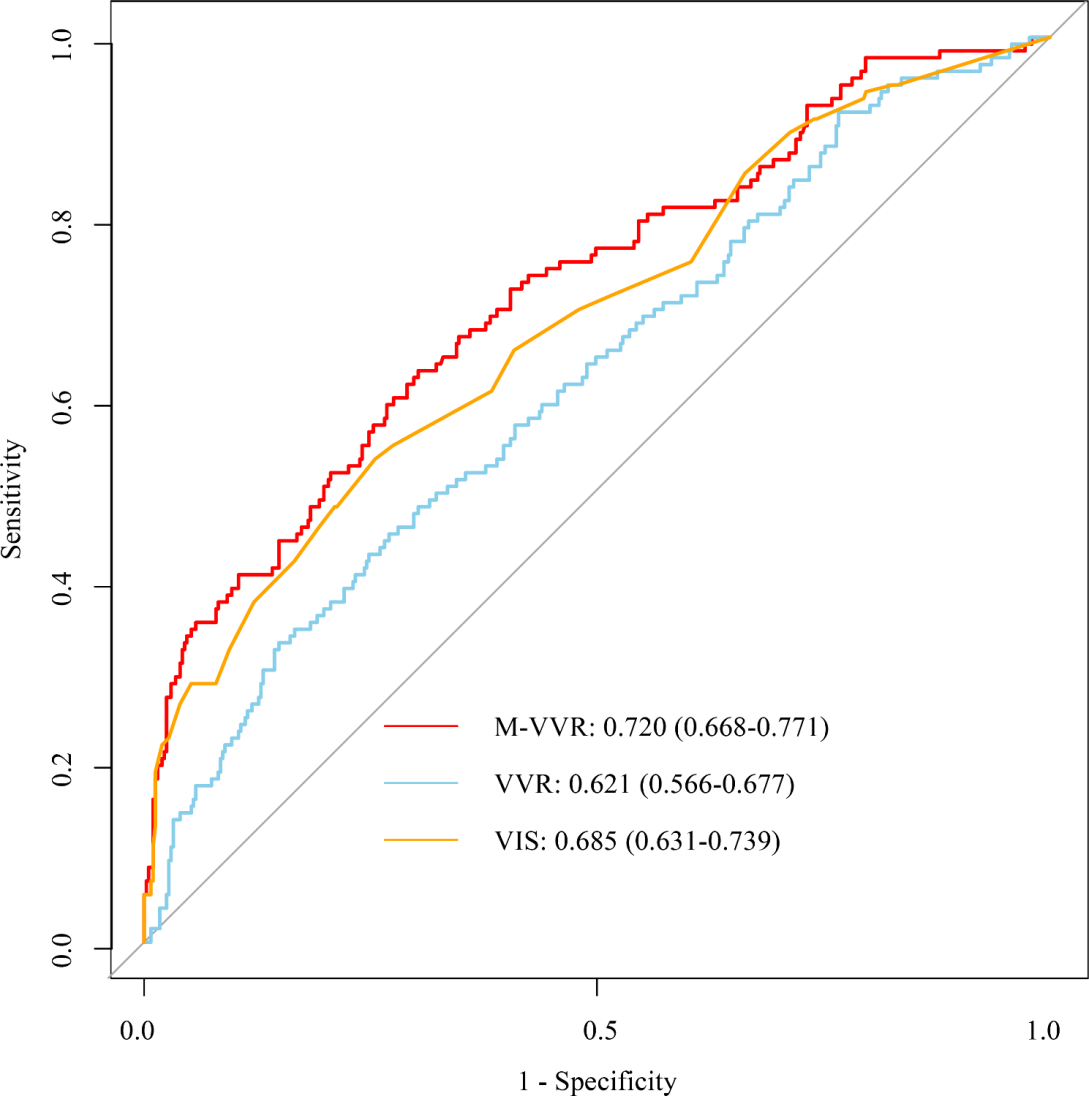
ROC curves of VIS, VVR, and M-VVR.

**Fig. 2:**
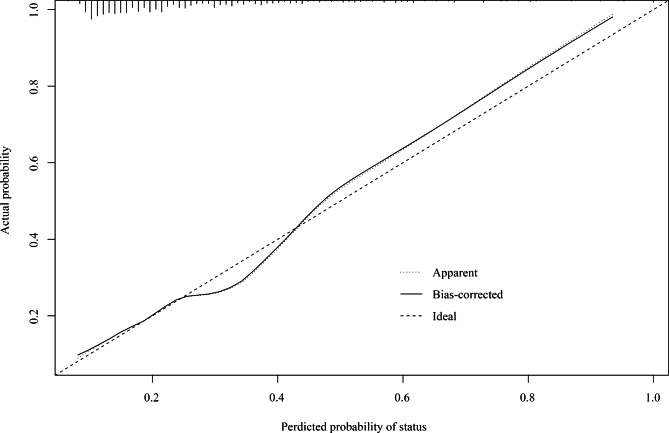
Calibration plot for M-VVR.

Figure 1 shows that ROC curves of VIS, VVR, and M-VVR predicting the poor prognosis of patients undergoing CABG. The AUC of M-VVR, VVR, and VIS was 0.720 (95%CI: 0.668–0.771), 0.621 (95%CI: 0.566–0.677), and 0.685 (95%CI: 0.631–0.739), respectively. Results of DeLong test displayed that the prediction performance of M-VVR was superior to VVR (*P* = 0.004) and VIS (*P* = 0.003) for the poor prognosis (Table 3). The calibration plot showed that M-VVR had a good calibration for the prediction of poor prognosis in patients undergoing CABG (Fig. 2).

**Table 3:** Comparison in the predictive performance of VIS, VVR, M-VVR

Variables	AUC	95% CI
M-VVR	0.720	0.668–0.771
VVR	0.621	0.566–0.677
VIS	0.685	0.631–0.739

VIS, vasoactive-inotropic score; VVR, vasoactive-ventilation-renal score; M-VVR, modified vasoactive-ventilation-renal score; AUC, the area under the receiver operating characteristic curve; CI, confidence interval

M-VVR vs. VVR: *P* = 0.004;

M-VVR vs. VIS: *P* = 0.003;

VVR vs. VIS: *P* = 0.043

### Comparing the performance of M-VVR with SinoSCORE

Supplementary table S2 shows that there was no association between SinoSCORE and the poor prognosis in patients undergoing CABG (OR: 1.20, 95%CI: 0.59–2.45; OR: 0.93, 95%CI: 0.49–1.78). Supplementary Fig. 1 shows the ROC curve of SinoSCORE predicting the poor prognosis of patients undergoing CABG. The AUC of SinoSCORE was 0.561 (95%CI: 0.508–0.613). DeLong test displayed that the prediction performance of M-VVR was superior to that of SinoSCORE (*P* < 0.001) (Supplementary table S3).

## Discussion

With the increasing trend of CABG in China, the prognosis of patients undergoing CABG becomes a common concern [4]. Many patients undergoing CABG suffer from poor prognosis, such as death, acute kidney injury, and heart failure [21, 22]. VIS has been reported as an independent factor for the mortality of patients after CABG [9]. VVR is an index based on VIS; although VVR has been reported in the congenital disease [13], its role remains unclear in the prognosis of patients undergoing CABG. M-VVR is a modified score for VVR, which is also not reported in CABG. In this study, we explored the performance of VIS, VVR, and M-VVR in predicting the poor prognosis of patients undergoing CABG. The results showed that higher VIS and M-VVR were associated with the increased odds of poor prognosis. The performance of M-VVR was superior to VIS and VVR in the prediction of poor prognosis.

Previous studies have confirmed that developing reliable scoring systems can be useful to predict the risk of poor prognosis [23, 24]. Ju et al. reported that age, creatinine, ejection fraction (ACEF) I score, and ACEF II score could be useful tools for prognostication after CABG [25]. In a Spanish cohort study, Leicester score (LS) was proven to be a valid score to identify the risk of acute kidney injury following cardiac surgery (CSA-AKI) [26]. Although these scores showed a good performance, they either ignored the pulmonary dysfunction or only focused on single outcome. M-VVR, an improvement on VVR, is a scoring system compositing VI, VIS, and Ccr, which used to assess cardiovascular, pulmonary, and renal functions [15]. In our study, M-VVR was confirmed to be positively associated with the poor prognosis of patients undergoing CABG. Previous studies have reported the positive association between VIS and poor prognosis in cardiac surgery [9, 27]. Kwon et al. manifested that increased postoperative VIS was independently correlated with the risk of 1-year mortality after CABG in adults [27]. Similarly, in this study, higher VIS was found to be associated with an increased odds of poor prognosis in patients undergoing CABG. Of the poor prognosis, the incidence of LCOS was near to 20% (97/537). Evidence has shown that the low LVEF was associated the high odds of LCOS [28]. In this study, LVEF of 29.8% of the patients was below the normal range.

The prediction performance of M-VVR was found to be superior to VIS and VVR in this study. M-VVR addresses three organ systems dysfunctions that most commonly affected by cardiac bypass surgery: cardiovascular, pulmonary, and renal, while VIS primarily measures the integrity of the cardiovascular system [11, 15]. The increased precision of M-VVR score compared to VIS may be explained by that M-VVR can capture the patients who have preserved hemodynamic integrity but have severe disease burden from postoperative lung or kidney damage. Miletic et al. has reported that adding measures of respiratory and renal dysfunction to the VIS is better to predict outcomes in cardiac surgery [29]. Both VVR and M-VVR were developed to measure cardiovascular, pulmonary, and renal functions, but the difference between them was the index used to assess renal function, which used ΔCr in VVR and Ccr in M-VVR. The reason for the superiority of M-VVR to VVR may be that Ccr is a better marker than ΔCr to estimate renal function [15]. ΔCr shows the change in postoperative serum creatinine from baseline [13]. Ge et al. have clarified that serum creatinine can be affected by age, diet, and change of muscle volume [30]. Compared to serum creatinine, Ccr decreases the impact of weight and age on outcomes, and is a quantitative indicator to measure renal damage due to it can reflect glomerular filtration rate and roughly evaluate the number of effective nephrons [30]. SinoSCORE is a scoring system developed by Chinese researchers and has been generally recognized as being able to predict the adverse prognosis after cardiac surgery [20, 31]. Compared to SinoSCORE, M-VVR showed the superior performance in the prediction of poor prognosis in this study, indicating that M-VVR may be a convincing tool to be used in the clinic for patients undergoing CABG. More studies are needed to further verify our findings.

There are some advantages in our study. First, we modify the VVR score. The serum creatinine needs to be tested only once (after the surgery) in M-VVR, while it needs to be tested twice (before and after the surgery) in VVR. Compared to VVR, M-VVR is a simpler and more convenient tool to be used in the clinic. Second, we exclude patients who is not the first time to undergo coronary artery intervention, which eliminates the impact caused by history of coronary artery intervention on the prognosis. Also, there are some limitations in our study. First, due to the limited sample size in death, mechanical circulatory support, stroke, and acute kidney injury, the prediction value of M-VVR for the single outcome cannot be explored. Second, we mainly explore the short-term (in-hospital) outcomes. Future study should concern on the prediction performance of M-VVR for the long-term outcomes of patients. Third, acute postoperative blood loss is also an important risk factor for the development of complications after heart surgery; however, due to serious data missing, we cannot include hemostasis-relevant variables in our analysis. Future studies should collect indicators of the hemostasis system and violations of this system to further verify our findings.

## Conclusion

In conclusion, we found that M-VVR score had a good performance in predicting the poor prognosis of patients undergoing CABG. Our findings indicated that a robust and easily calculated disease severity score may be developed in our study to predict the outcomes of patients undergoing CABG.

## Electronic supplementary material

Below is the link to the electronic supplementary material.

Supplementary Material 1

Supplementary Material 2

Supplementary Material 3

## Data Availability

The datasets used and/or analyzed during the current study are available from the corresponding author on reasonable request.

## Abbreviations

CABG: Coronary artery bypass grafting
CAD: Coronary artery disease
VIS: Vasoactive-inotropic score
VVR: Vasoactive-ventilation-renal
Ccr: Creatinine clearance
VI: Ventilation index
LCOS: Low cardiac output syndrome
BMI: Body mass index
LVEF: Left ventricular ejection fraction
SinoSCORE: Sino System for Coronary Operative Risk Evaluation
Mean ± SD: Mean ± standard deviation
M (Q1, Q3): Median and interquartile range
OR: Odds ratios
CIs: Confidence intervals
ROC: Receiver operating characteristics
AUC: Area under the ROC curve
