# Supplementary material for: Comparison of vasoactive-inotropic score, vasoactive-ventilation-renal score, and modified vasoactive-ventilation-renal score for predicting the poor prognosis after coronary artery bypass grafting
Source: BMC Cardiovasc Disord. 2023 May 24;23:274. doi: 10.1186/s12872-023-03313-9 (PMC10210316; doi:10.1186/s12872-023-03313-9)
Supplement: Supplementary file 1 — Supplementary Material 1 [file 12872_2023_3313_MOESM1_ESM.docx]

**VIS calculation formula:** dopamine dose (μg/kg/min) + dobutamine dose (μg/kg/min) + 10 × milrinone dose (μg/kg/min) + 100 × epinephrine dose (μg/kg/min) + 100 × norepinephrine dose (μg/kg/min) + 10,000 × vasopressin dose (μg/kg/min) [1]. VIS was recorded as zero for patients not receiving vasoactive support during the blood gas measurement.

**VVR calculation formula:** ventilation index (VI) + VIS + ΔCr × 10 [2]. VI was calculated as [respiratory rate × (peak inspiratory pressure - positive end expiratory pressure) × partial pressure of arterial CO_2_]/1,000 [1, 2]. VI was equal to zero for patients with no mechanical ventilation [1, 2]. ΔCr was calculated as postoperative serum creatinine measurements (postoperative 24h) subtracted preoperative serum creatinine measurements (preoperative 24-48h), and it was recorded as zero for those who postoperative serum creatinine less than or equal to the baseline [1, 2].

**M-VVR calculation formula:** VI + VIS + Ccr. Ccr was calculated as the following [3]:

Ccr (mL/min) = $\frac{\left（ 140 - age \right） \times weight (kg)}{72 \times serum creatinine (mg/dL)}$ (for male);

Ccr (mL/min) = $\frac{\left（ 140 - age \right） \times weight (kg)}{85 \times serum creatinine (mg/dL}$ (for female).

**References**

1. Cashen K, Costello JM, Grimaldi LM, Narayana Gowda KM, Moser EAS, Piggott KD, et al. Multicenter Validation of the Vasoactive-Ventilation-Renal Score as a Predictor of Prolonged Mechanical Ventilation After Neonatal Cardiac Surgery. Pediatric critical care medicine : a journal of the Society of Critical Care Medicine and the World Federation of Pediatric Intensive and Critical Care Societies. 2018; 19: 1015-23.

2. Scherer B, Moser EA, Brown JW, Rodefeld MD, Turrentine MW, Mastropietro CW. Vasoactive-ventilation-renal score reliably predicts hospital length of stay after surgery for congenital heart disease. The Journal of thoracic and cardiovascular surgery. 2016; 152: 1423-9.e1.

3. Ge J, Jin Z, Feng X, Pan W, Liu L, Wu M, et al. Creatinine clearance rate predicts prognosis of patients with systemic lupus erythematosus: a large retrospective cohort study. Clinical rheumatology. 2021; 40: 2221-31.
