# Supplementary material for: Comparison of vasoactive-inotropic score, vasoactive-ventilation-renal score, and modified vasoactive-ventilation-renal score for predicting the poor prognosis after coronary artery bypass grafting
Source: BMC Cardiovasc Disord. 2023 May 24;23:274. doi: 10.1186/s12872-023-03313-9 (PMC10210316; doi:10.1186/s12872-023-03313-9)
Supplement: Supplementary file 2 — Supplementary Material 2 [file 12872_2023_3313_MOESM2_ESM.docx]

**Supplementary table S1 Number and percentage of each complication**

| **Variables** | **n (%)** |
| --- | --- |
| Death |  |
| No | 530 (98.70) |
| Yes | 7 (1.30) |
| Cardiopulmonary resuscitation |  |
| No | 526 (97.95) |
| Yes | 11 (2.05) |
| Mechanical circulatory support |  |
| No | 536 (99.81) |
| Yes | 1 (0.19) |
| LCOS |  |
| No | 440 (81.94) |
| Yes | 97 (18.06) |
| Stroke |  |
| No | 528 (98.32) |
| Yes | 9 (1.68) |
| Acute kidney injury |  |
| No | 536 (99.81) |
| Yes | 1 (0.19) |
| Nervous system damage |  |
| No | 502 (93.48) |
| Yes | 35 (6.52) |

LCOS, low cardiac output syndrome

**Supplementary table S2 Univariate and multivariate analyses for** **the association between SinoSCORE and poor prognosis**

| **Variables** | **Model 1** | | **Model 2** | |
| --- | --- | --- | --- | --- |
|  | **OR (95% CI)** | ***P*** | **OR (95% CI)** | ***P*** |
| SinoSCORE |  |  |  |  |
| ≤ 1 | Ref |  | Ref |  |
| 2-5 | 0.81 (0.46-1.41) | 0.455 | 1.20 (0.59-2.45) | 0.619 |
| ≥ 6 | 1.43 (0.92-2.22) | 0.107 | 0.93 (0.49-1.78) | 0.830 |

SinoSCORE, Sino System for Coronary Operative Risk Evaluation; OR, odds ratio; CI, confidence interval.

Model 1, unadjusted model;

Model 2, adjusted for gender, BMI, hypertension, diabetes, surgery methods, and LVEF.

**Supplementary table S3 Comparison in the predictive performance between M-VVR and SinoSCORE**

| **Variables** | **AUC** | **95% CI** |
| --- | --- | --- |
| M-VVR | 0.720 | 0.668-0.771 |
| SinoSCORE | 0.561 | 0.508-0.613 |

SinoSCORE, Sino System for Coronary Operative Risk Evaluation; AUC, the area under the receiver operating characteristic curve; CI, confidence interval.

M-VVR *vs*. SinoSCORE: *P* < 0.001;
