# Supplementary figures and images for: Comparison of vasoactive-inotropic score, vasoactive-ventilation-renal score, and modified vasoactive-ventilation-renal score for predicting the poor prognosis after coronary artery bypass grafting
Source: BMC Cardiovasc Disord. 2023 May 24;23:274. doi: 10.1186/s12872-023-03313-9 (PMC10210316; doi:10.1186/s12872-023-03313-9)

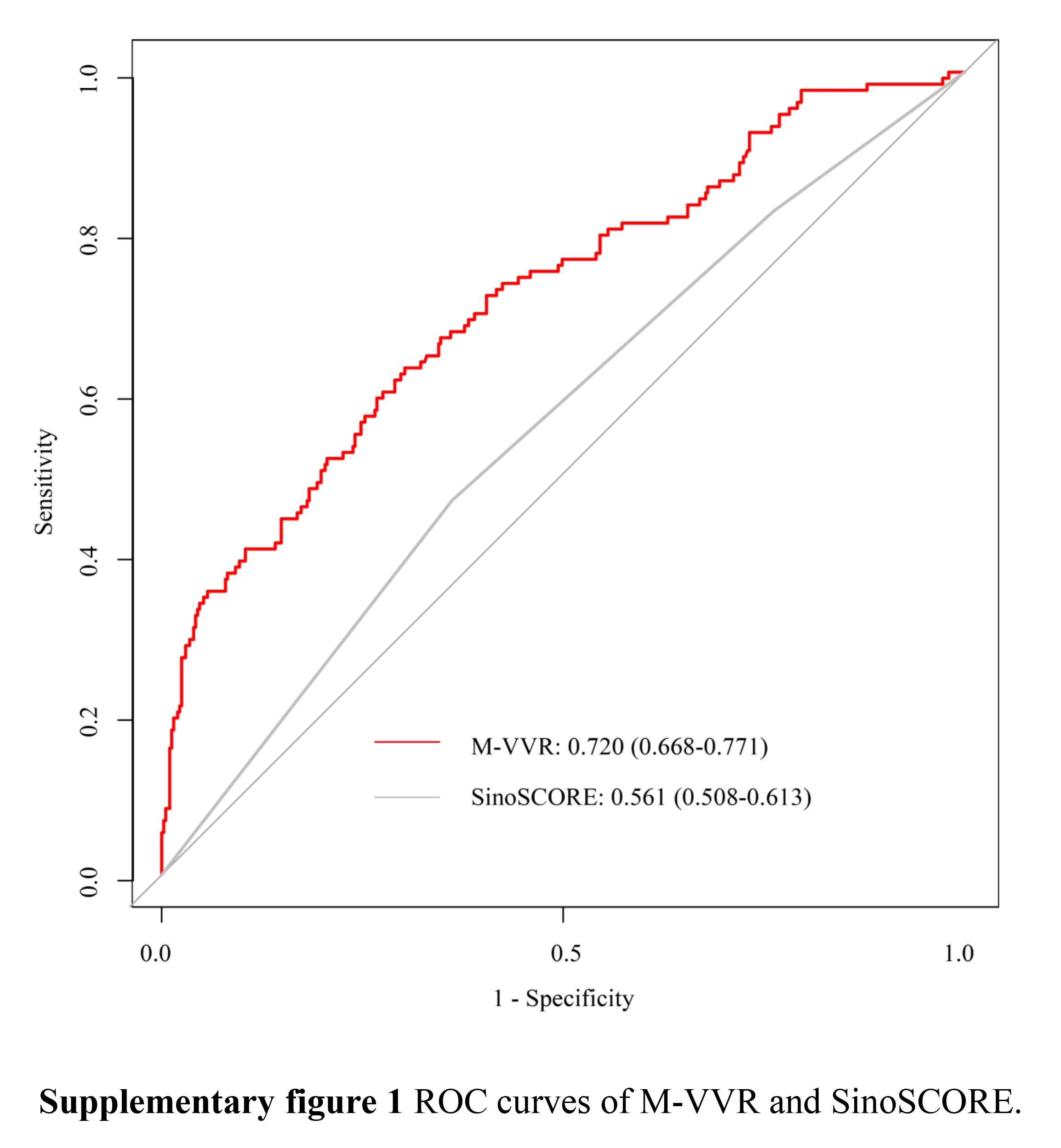

Supplement: Supplementary file 3 — Supplementary Material 3 [file 12872_2023_3313_MOESM3_ESM.tif]
